# Breeding history, not dietary adaptation, drives growth of house crickets, *Acheta domesticus* (Orthoptera: Gryllidae), on novel feeds

**DOI:** 10.1093/jisesa/ieag122

**Published:** 2026-11-12

**Authors:** Ludvig Lindfors Frimodig, Matthew Low, Joachim R de Miranda, Åsa Berggren

**Affiliations:** Department of Ecology, Swedish University of Agricultural Sciences, Uppsala, Sweden; Department of Ecology, Swedish University of Agricultural Sciences, Uppsala, Sweden; Department of Ecology, Swedish University of Agricultural Sciences, Uppsala, Sweden; Department of Ecology, Swedish University of Agricultural Sciences, Uppsala, Sweden

**Keywords:** insect nutrition, dietary history, feed adaptation, phenotypic plasticity, insect rearing

## Abstract

Understanding how insect populations respond to novel diets is key to optimizing feed formulations and production efficiency in insect farming. In a controlled, replicated feeding trial, we reciprocally cross-fed 2 house cricket (*Acheta domesticus* (L.)) lines that had been maintained for >4 yr on distinct diets, a cereal‑based mix and a commercial formulation; the commercial-fed line had also been selected for larger body size. We partitioned growth into effects of diet adaptation (the advantage of the historical diet), diet composition and body-size selection. Mean body mass increased in all cages, and there was no evidence that survival differed among treatments. Line identity dominated growth: the commercial-fed line was more than twice as heavy at week 5 as the cereal-fed line, irrespective of feed (0.19 g; 95% CrI 0.12 to 0.25). The higher-fat commercial diet probably increased mass slightly in both lines (0.03 g; 95% CrI −0.02 to 0.10; Pr(effect > 0) = 0.87). In contrast, there was no evidence of a home-diet advantage on either an absolute or a proportional scale (0.003 g; 95% CrI −0.06 to 0.07; Pr = 0.55). Switching to a novel diet carried no detectable growth cost, suggesting physiological plasticity rather than diet adaptation. For producers, these results imply that line origin matters far more than the choice between these feeds. Future work should use replicated lines, follow crickets to harvest age at production densities, and combine feed‑intake measurements with microbiome analyses to separate consumption from conversion efficiency effects.

## Introduction

To enhance circular resource use and improve the sustainability of insect rearing systems, a growing number of alternative feeds are being tested. These often consist of byproducts from the food and feed industries, whose nutrients would otherwise go unused (Miech et al. 2016, Kuo and Fisher 2022). Although studies have examined how some insect species respond to such novel diets (e.g. Acur et al. 2024, Gourgouta et al. 2024), many species-byproduct combinations remain unexplored. In addition, little is known about how long-term dietary history across generations influences an insect’s capacity to process and assimilate nutrients from different feeds. Understanding these responses is important for developing sustainable feeds and productive insect populations and for interpreting feeding trials using populations maintained for generations on standard rearing diets (Berggren et al. 2019).

Insects harbor diverse and dynamic gut microbiota that contribute to digestion, detoxification and nutrient assimilation. These microbial communities can shift rapidly in response to dietary inputs and may also adapt alongside the host over time. In crickets, diet and rearing environment can shape gut microbial communities, and gut bacteria contribute to carbohydrate use and nutrient processing (Kaufman and Klug 1991, Santo Domingo et al. 1998, Ng et al. 2018). Studies on other insects have found that microbiota can contribute to adaptation to nutritionally challenging or novel diets, with hosts dependent on microbiota for poor diets and microbiome-mediated host-plant adaptation (Sudakaran et al. 2015, Zhang et al. 2024). These findings suggest that microbial plasticity may contribute to responses to novel feeds, although this needs to be tested directly.

To address this limited understanding of how species respond to novel feeds, we conducted a controlled feeding trial using 2 lines of house cricket (*Acheta domesticus* (L.)) that had been maintained for more than 4 yr on distinct diets: a cereal-based diet and a commercial diet formula. The commercial-fed line also had a history of selection for larger body size as part of a breeding experiment, which was considered when interpreting line-level differences. These long-term lines, referred to as “cereal-fed” and “commercial-fed,” were reciprocally cross-fed to evaluate their performance on both ancestral and novel diets. House crickets achieve roughly two-thirds of their adult dry mass within the first 28 d at 30 °C, with later instars involving mainly cuticle maturation rather than new biomass deposition (Clifford and Woodring 1990), making early development a sensitive window for detecting diet-induced differences in growth. We tested 2 expectations, independent of possible size differences resulting from selection in one line: if responses were primarily plastic, the effect of switching diet should be similar in both lines, with no advantage of the ancestral diet; if long-term adaptation had occurred, each line should perform best on its ancestral diet. Because each line was tested on both diets, this comparison is independent of any general difference in nutritional quality between feeds. Microbial mechanisms were considered in our interpretation, but were not measured directly. The experiment therefore evaluates whether growth responses are more consistent with phenotypic plasticity or diet adaptation, rather than definitively distinguishing between them.

## Material and Methods

*A. domesticus* individuals were gathered from wild populations in Sweden and maintained under standard rearing conditions for over 4 yr. Two lines originating from a shared ancestral stock were reared on different diets during this period: one on cereal and one on commercial feed. During the >4 yr of separate rearing, the commercial-fed line was also subjected to phenotypic selection aimed at increasing body size, using a combination of morphological and other phenotypic traits for breeding selection, whereas no corresponding selection was applied to the cereal-fed line. The cereal-fed line was maintained on a lab-produced standard diet consisting of 308 g/kg wheat bran, 296 g/kg oat bran, 224 g/kg wheat flour, 150 g/kg rapeseed meal, 17.9 g/kg calcium carbonate and 4.0 g/kg vitamin-mineral premix on a dry-matter basis (19.2% crude protein, 2.5% crude fat, Vaga et al. 2020). The commercial-fed line received a 50:50 mixture by mass of pelleted alfalfa (15.0% crude protein, 3.5% crude fat) and poultry feed (16.5% crude protein, 5.5% crude fat) (Svenska Foder AB, Lidköping, Sweden). The feeds were not nutritionally matched, because the aim was to compare the diets on which the 2 lines had actually been maintained. For the experiment, 48 juvenile crickets (between 2 and 3 wk old) were randomly selected from each line (juveniles from each line hatched at approximately the same time, giving 96 individuals of similar age for the experiment), and assigned to 1 of 4 treatment groups: (i) cereal-fed line on cereal diet (ancestral), (ii) cereal-fed line on the commercial diet (novel), (iii) commercial-fed line on commercial diet (ancestral), and (iv) commercial-fed line on the cereal diet (novel). Each treatment was replicated across 4 cages (16 cages in total), each starting with 6 individuals. Baseline mean individual body mass per cage was recorded at the start of the trial using the same group-weighing procedure and is reported for each line × diet combination in Supplementary Table S1. Feed was provided *ad libitum*, and all crickets were reared under standard rearing conditions, at approximately 30 °C and 40% relative humidity. Survival and growth were recorded at the start of the trial, and then weekly to week 5 (4 wk of feeding). Limiting the trial to this rapid-growth window reduces confounding effects of final-instar maturation while capturing >70% of the variation in ultimate harvest mass (Vaga et al. 2021). Survival was measured by counting live individuals per cage. Growth was assessed by weighing all remaining crickets in each cage on a precision balance (ML1602T/00 Mettler-Toledo, 0.01 g precision), subtracting container mass and dividing by the number of surviving individuals to estimate mean individual body mass per cage. Cage was therefore treated as the experimental unit because individuals within a cage shared the same feeding and rearing environment and were not weighed independently. Extraneous material (e.g. feed remains, frass) was removed before weighing to ensure only cricket mass was recorded.

### Statistical Analysis

The response was mean individual body mass per cage at week 5 (*n* = 16 cages). Diet adaptation, diet composition and body-size selection were derived as contrasts of the posterior cell means: ancestral minus novel diet, commercial minus cereal diet, and commercial-fed minus cereal-fed line, each averaged over the other factor. Because each line was fed both diets, a general difference in nutritional quality between feeds appears in the diet composition contrast, whereas diet adaptation is estimated from the line × diet interaction and is independent of which feed is richer. These models were implemented in a Bayesian framework in R (R Core Team 2026) using JAGS (Plummer 2003) with minimally informative priors (normal: mean 0, precision 0.001 for beta terms; uniform: 0, 1 on the residual SD). This allowed us to not only estimate the body mass for each group, but also derive posterior distributions of all between-group differences to compare the relative impact on body mass of each effect, and the probability of one group having a larger effect than another (values near 0.5 indicate no support for an effect in either direction, and values near 0 or 1 indicate strong support for a negative or positive effect; we interpret these probabilities on a graded scale rather than against fixed thresholds). Bayesian models were checked for convergence by visual inspection of stability and mixing of the chains and fit using posterior predictive checks; posteriors were determined from 10,000 samples. To check whether conclusions depended on the scale of measurement, given the size difference between lines, we refitted the model to log-transformed cage mean mass; back-transformed contrasts are ratios of geometric means (Supplementary Table S2). We also derived the posterior difference between the body-size selection and diet adaptation effects. Survival was analyzed using Kaplan–Meier estimation to describe the probability of survival at each weekly time point. To compare survival between lines (cereal-fed vs. commercial-fed) and between diets (cereal vs. commercial), log-rank tests were used. To account for the non-independence of individuals within cages, Cox proportional hazards models were fitted with a gamma frailty term for cage. All survival analyses were conducted using the survival package (Therneau 2026) in R.

## Results

Mean body mass increased in all cages during the trial, indicating that both diets supported growth. The absolute gains we report are in the same order as full-cycle studies that continue to day 60 to 65 (e.g. Oonincx et al. 2015), suggesting that our truncated design did not systematically under- or over-estimate performance. However, growth patterns varied substantially between lines. At the start of the trial, the size-selected line was lighter than the cereal-fed line (mean ± SD individual mass per cage 0.035 ± 0.007 vs. 0.045 ± 0.008 g), and initial mass differed little between diet treatments within lines (≤ 0.004 g; Supplementary Table S1). The commercial-fed line, which had a history of selection for increased body size, reached a much higher final mean weight (0.33 ± 0.04 g) than the cereal-fed line (0.13 ± 0.01 g), about 2.3 times heavier (ratio 2.34; 95% CrI 1.87 to 2.92) (Fig. 1). The greater mass of the size-selected line at week 5 therefore reflects faster growth during the trial, an approximately 9-fold vs. 3-fold increase in mass, rather than a larger starting size.

**Fig. 1.**
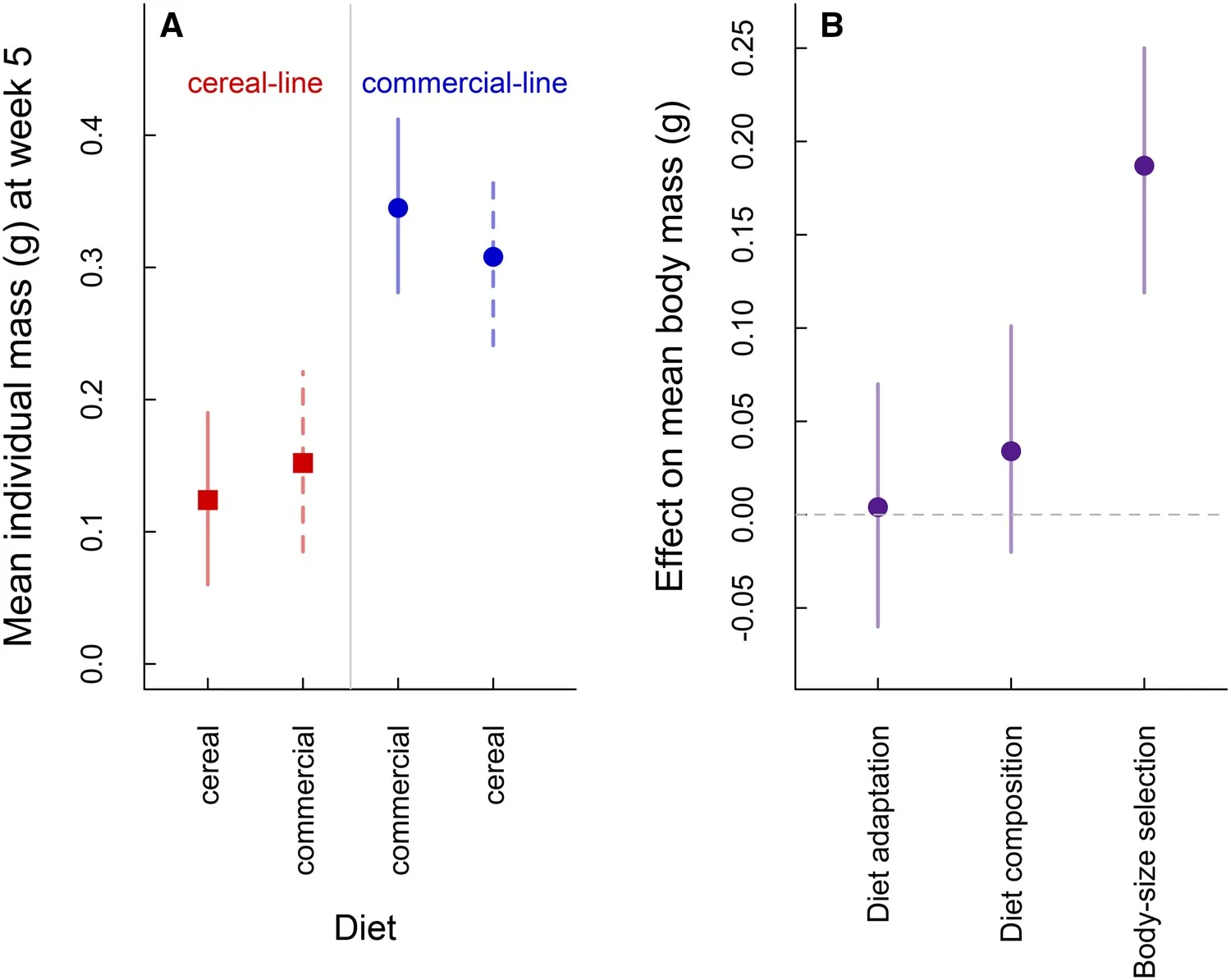
Growth of 2 house cricket (*Acheta domesticus*) lines reciprocally cross-fed a cereal-based diet and a commercial diet. A) Mean individual body mass (g) at week 5 of the cereal-fed line (red squares) and the size-selected, commercial-fed line (blue circles) on each diet. Solid bars denote the line’s ancestral diet and dashed bars the novel diet. B) Estimated effects (g) on mean individual body mass at week 5 of diet adaptation (ancestral minus novel diet), diet composition (commercial minus cereal diet) and body-size selection (size-selected commercial-fed line minus cereal-fed line), each averaged over the other factor. The dashed horizontal line marks no effect. In both panels, points show posterior means and bars show 95% credible intervals from a Bayesian linear model fitted to cage means (*n* = 4 cages of 6 crickets per line × diet combination). Estimates for (A) are given in Supplementary Table S1 and for (B) in Supplementary Table S2.

Both lines were heavier on the commercial diet: the commercial-fed line reached 0.35 ± 0.04 g on the commercial diet and 0.31 ± 0.02 g on the cereal diet, and the cereal-fed line 0.15 ± 0.01 g and 0.12 ± 0.01 g, respectively. There was no evidence of a home-diet advantage (diet adaptation 0.003 g; 95% CrI −0.06 to 0.07; Pr(effect > 0) = 0.55), a probable small positive effect of the commercial diet (diet composition 0.033 g; 95% CrI −0.02 to 0.10; Pr = 0.87) and a strong effect of body-size selection (0.188 g; 95% CrI 0.12 to 0.25; Pr > 0.99) (Fig. 1B; Supplementary Table S2). The diet adaptation effect was smaller than the effect of body-size selection (difference 0.18 g; 95% CrI 0.09 to 0.28; Pr > 0.999). Conclusions were unchanged on a log scale: crickets on their historical diet were about 6% lighter than on the novel diet (ratio 0.94; 95% CrI 0.75 to 1.17; Pr = 0.29), the commercial diet increased mass by about 16% (1.16; 0.94 to 1.44; Pr = 0.92), and the commercial-fed line was 2.3 times heavier (2.34; 1.87 to 2.92) (Supplementary Table S2). Although most cages (11 of 16) recorded some mortality, overall survival was generally high across all treatments (Supplementary Fig. S1; Supplementary Table S3). Kaplan–Meier estimated survival to week 5 was 85.4% (cereal-fed line) and 79.2% (commercial-fed line), and 81.2% (cereal diet) and 83.3% (commercial diet). There was no evidence that survival differed between lines (log-rank χ^2^(1) = 0.79, *P* = 0.37) or between diets (χ^2^(1) = 0.06, *P* = 0.81). Cox frailty models accounting for within-cage clustering confirmed these findings (line: HR = 1.52, 95% CI: 0.58 to 4.00, *P* = 0.40; diet: HR = 0.89, 95% CI: 0.32 to 2.45, *P* = 0.82), though results should be interpreted with caution given the low number of mortality events (17 deaths across 96 individuals). There was also no evidence that survival differed between ancestral and novel diets (log-rank χ^2^(1) = 0.07, *P* = 0.79; HR = 1.13, 95% CI 0.41 to 3.10, *P* = 0.81), and Cox models without the frailty term gave nearly identical estimates (Supplementary Table S3).

## Discussion

This study shows that *A. domesticus* lines with different dietary and selection histories differed in growth, with the observed patterns providing no evidence of feed-specific adaptation. The commercial-fed line grew more and faster than the cereal-fed line on both diets, being about 2.3 times heavier at week 5, despite starting the trial about 20% lighter. This difference is most plausibly explained by the commercial-fed line’s history of selection for larger body size. Survival was generally high during the experiment, and there was no evidence that survival differed between lines or diets, suggesting that the growth differences observed are unlikely to reflect differential mortality acting on cage-level weight estimates.

The feeds differed in both ingredient identity and macronutrient composition: the cereal feed contained slightly more protein (19.2%) but less fat (2.5%) than the mixed commercial feeds (mixture protein 15.8%; fat 4.5%) (Vaga et al. 2020). Both lines probably grew better on the higher-fat commercial diet (Pr = 0.87 on the absolute scale and 0.92 on the proportional scale), suggesting that feed composition contributed to growth differences. The slightly higher mass of the commercial-fed line on its ancestral diet therefore reflects the same diet composition effect seen in the cereal-fed line rather than an ancestral-diet advantage. On a proportional scale, the cereal-fed line, for which the commercial diet was novel, responded more strongly (+24% vs. +10%). Together, these patterns provide no evidence of diet-specific adaptation and are compatible with phenotypic plasticity.

Our findings therefore point to physiological plasticity as a likely contributor to variation in weight gain across diets, while microbial plasticity remains a plausible but untested mechanism. The cereal-fed line’s slightly higher growth on the commercial diet suggests that crickets with a long history on a cereal diet can nevertheless exploit a higher-fat feed, while the commercial-fed line showed only a modest reduction in growth when switched to the cereal diet. Switching to a novel diet thus carried no detectable growth cost in either line, on an absolute or a proportional scale. Notably, the commercial-fed line was selected for growth on the commercial diet, which would be expected to favor specialization to that feed, yet it showed no home-diet advantage. This pattern is consistent with feed composition contributing to performance independently of diet familiarity. Plasticity in nutrient assimilation could arise through host-level physiological adjustments or changes in the gut microbiome. Although microbial responses were not measured, previous work in crickets has shown that diet and rearing environment can alter gut microbial communities and that hindgut bacteria can contribute to dietary carbohydrate utilization (Kaufman and Klug 1991, Santo Domingo et al. 1998, Ng et al. 2018). Microbiome-mediated responses therefore remain a plausible mechanism for future investigation but cannot be inferred from the present experiment.

The main limitation of our study is that each dietary history was represented by a single line, so line differences cannot be formally separated from genetic drift; drift, however, acts in a random direction, whereas the observed difference matches the direction of selection. Because the trial was short, small and cage-based at a modest scale, the results should be interpreted as broad growth patterns rather than as precise production-level estimates, and the possibility of a small home-diet advantage (<0.07 g) cannot be completely excluded. Because the diets differed in several nutrients and ingredients, we cannot identify which feed properties underlie the diet composition effect, although the reciprocal design separates this effect from feed familiarity. Interpretation is further limited by the lack of feed-intake data, which prevents separation of feed consumption from conversion efficiency, a distinction that matters for production; the size-selected line, for instance, may simply eat more. Directly testing diet-specific adaptation will require replicated lines for each dietary history, maintained for multiple generations in a common garden without continued size selection, whereas quantifying feed intake on iso-energetic, iso-protein, and iso-fat diets, for example in intake-matched or pair-fed designs, would identify which feed properties drive growth. Our results highlight the importance of selection background and to a lesser extent feed composition in shaping cricket growth (Berggren et al. 2018); for producers, line origin appears to matter more than the choice between these feeds.

## Supplementary Material

ieag122_Supplementary_Data
