# Supplementary material for: Breeding history, not dietary adaptation, drives growth of house crickets, Acheta domesticus (Orthoptera: Gryllidae), on novel feeds
Source: J Insect Sci. 2026 Nov 12;26(6):ieag122. doi: 10.1093/jisesa/ieag122 (PMC13755863; doi:10.1093/jisesa/ieag122)
Supplement: Supplementary file 1 [file ieag122_supplementary_data.zip › ieag122_Supplementary Files/Figure_S1.pdf]

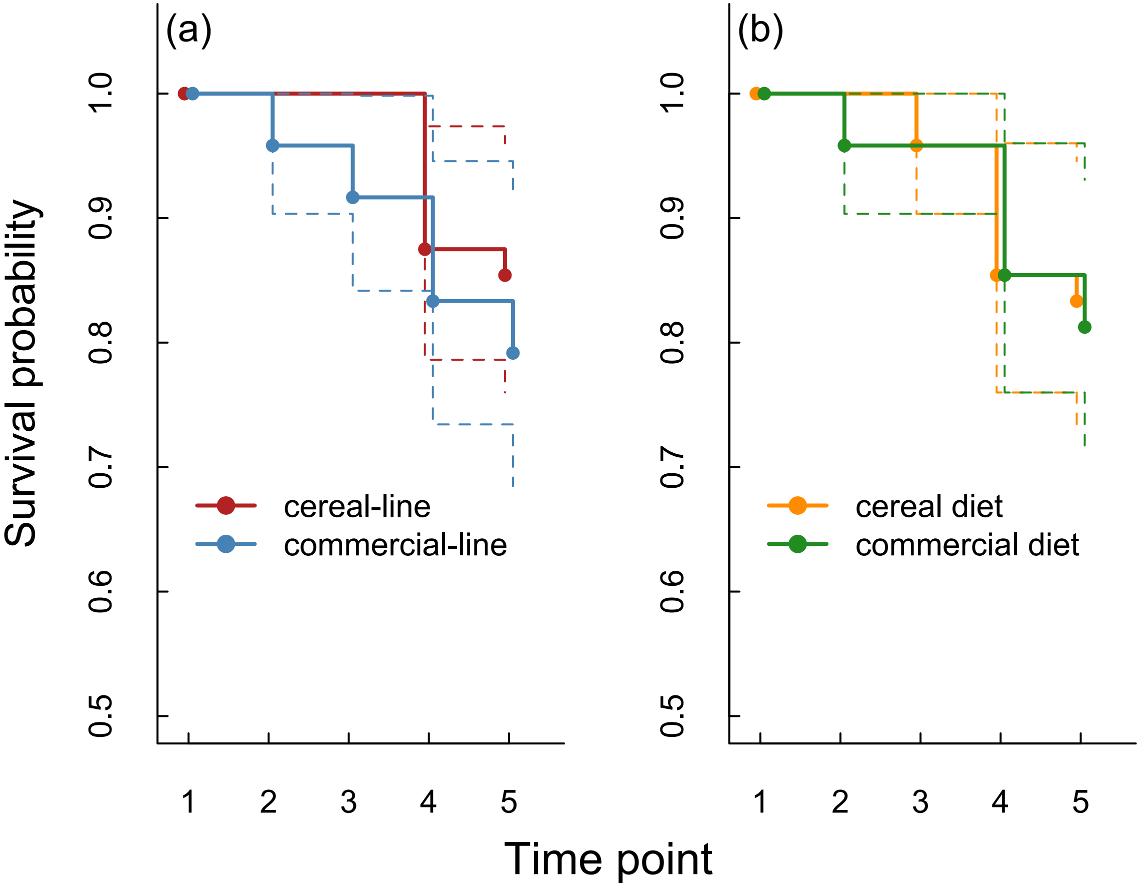

Fig. S1. Kaplan–Meier survival of house crickets (*Acheta domesticus*) from the start of the trial (week 1) to week 5. (a) By line, pooled across diets: cereal-fed line (red) and size-selected, commercial-fed line (blue). (b) By diet, pooled across lines: cereal diet (orange) and commercial diet (green). Solid lines and points show the survival estimate at each weekly time point, and dashed lines show 95% confidence intervals. Each curve is based on 48 individuals in eight cages (96 individuals and 17 deaths in total). Curves are offset slightly along the x-axis for clarity. There was no evidence that survival differed between lines or between diets (Table S3).
