# Supplementary material for: Breeding history, not dietary adaptation, drives growth of house crickets, Acheta domesticus (Orthoptera: Gryllidae), on novel feeds
Source: J Insect Sci. 2026 Nov 12;26(6):ieag122. doi: 10.1093/jisesa/ieag122 (PMC13755863; doi:10.1093/jisesa/ieag122)
Supplement: Supplementary file 1 [file ieag122_supplementary_data.zip › ieag122_Supplementary Files/Supplementary Tables.pdf]

Supplementary Tables

Table S1. Mean individual body mass (in grams) per cage at the start of the trial (baseline) and at week 5 for each line on each diet, with the within-line effect of the commercial relative to the cereal diet. Each line's ancestral diet is given in brackets. Baseline values are means ± SD across cages (n = 4 cages per line × diet). Week-5 values and contrasts are posterior means with 95% credible intervals from the Bayesian linear model (see Table S2); Pr(effect > 0) is the proportion of the posterior distribution above zero from the commercial – cereal contrast at week 5 (i.e. the probability that growth on the commercial diet > growth on the cereal diet).

Line	Baseline cereal	Baseline commercial	Week5 cereal	Week5 commercial	Commercial − cereal diet (g)	Pr(effect > 0)
Cereal-fed (ancestral: cereal)	0.047 ± 0.009	0.043 ± 0.008	0.124 (0.059-0.189)	0.154 (0.089-0.220)	0.030 (-0.063-0-121)	0.75
Size-selected, commercial-fed (ancestral: commercial)	0.037 ± 0.008	0.034 ± 0.006	0.308 (0.241-0.375)	0.347 (0.281-0.421)	0.039 (-0.053-0.134)	0.81

Table S2. Posterior estimates of the effects of diet adaptation, diet composition and body-size selection on mean individual body mass (in grams) per cage at week 5, on the absolute and proportional scales. Diet adaptation is the advantage of the ancestral over the novel diet; diet composition, the advantage of the commercial over the cereal diet; and body-size selection, the advantage of the size-selected (commercial-fed) line over the cereal-fed line, each averaged over the other factor (n = 4 cages per line × diet). Absolute effects are posterior means ± SD of between-group differences. Proportional effects are back-transformed ratios of geometric means (posterior medians) from the same model fitted to log-transformed mass. Pr(effect > 0) is the proportion of the posterior distribution above zero (above 1 for ratios). The last row is the posterior difference between the body-size selection and diet adaptation effects.

Effect	Absolute: mean ± SD	95% CrI	Pr(effect > 0)	Proportional: ratio	95% CrI	Pr(effect > 0)
Diet adaptation	0.003 ± 0.033	−0.06 to 0.07	0.55	0.94	0.75–1.17	0.29
Diet composition	0.033 ± 0.032	−0.02 to 0.10	0.87	1.16	0.94–1.44	0.92
Body-size selection	0.188 ± 0.033	0.12 to 0.25	> 0.99	2.34	1.87–2.92	> 0.99
Selection minus adaptation	0.183 ± 0.049	0.09 to 0.28	> 0.99	–	–	–

Table S3. Survival of house crickets to week 5 by line and diet. (a) Deaths and Kaplan-Meier survival; with no censoring, survival equals the proportion of individuals alive at week 5. (b) Comparisons between lines and between diets using log-rank tests and Cox proportional hazards models with and without a gamma frailty term for cage (17 deaths across 96 individuals).

(a)

Line	Diet	Individuals at start	Deaths	Survival to week 5 (%)
Cereal-fed	Cereal (ancestral)	24	4	83.3
Cereal-fed	Commercial (novel)	24	3	87.5
Cereal-fed	Both diets	48	7	85.4
Size-selected, commercial-fed	Commercial (ancestral)	24	5	79.2
Size-selected, commercial-fed	Cereal (novel)	24	5	79.2
Size-selected, commercial-fed	Both diets	48	10	79.2
Both lines	Cereal diet	48	9	81.2
Both lines	Commercial diet	48	8	83.3
Both lines	Ancestral diet	48	9	81.2
Both lines	Novel diet	48	8	83.3
All	All	96	17	82.3

(b)

Comparison	Log-rank $\chi^2(1)$, p	Cox with frailty: HR (95% CI), p	Cox without frailty: HR (95% CI), p
Line (size-selected vs. cereal-fed)	0.79, 0.37	1.52 (0.58–4.00), 0.40	1.52 (0.58-3.98), 0.40
Diet (commercial vs. cereal)	0.06, 0.81	0.89 (0.32–2.45), 0.82	0.89 (0.34-2.31), 0.81
Diet history (ancestral vs. novel)	0.07, 0.79	1.13 (0.41–3.10), 0.81	1.13 (0.44-2.93), 0.80
